# Particle Beam Therapy Tolerance and Outcome on Patients with Autoimmune Diseases: A Single Institution Matched Case–Control Study

**DOI:** 10.3390/cancers13205183

**Published:** 2021-10-15

**Authors:** Giulia Riva, Barbara Vischioni, Sara Gandini, Stefano Cavalieri, Sara Ronchi, Amelia Barcellini, Maria Bonora, Agnieszka Chalaszczyk, Rossana Ingargiola, Viviana Vitolo, Maria Rosaria Fiore, Alberto Iannalfi, Ester Orlandi

**Affiliations:** 1 Clinical Department, National Center for Oncological Hadrontherapy (CNAO), 27100 Pavia, Italy; Barbara.Vischioni@Cnao.it (B.V.); ronchi@cnao.it (S.R.); barcellini@cnao.it (A.B.); bonora@cnao.it (M.B.); Chalaszczyk@cnao.it (A.C.); rossana.ingargiola@cnao.it (R.I.); vitolo@cnao.it (V.V.); mariarosaria.fiore@cnao.it (M.R.F.); alberto.iannalfi@cnao.it (A.I.); ester.orlandi@cnao.it (E.O.); 2 Department of Experimental Oncology, European Institute of Oncology (IEO), IRCCS, 20139 Milan, Italy; sara.gandini@ieo.it; 3 Head and Neck Cancer Medical Oncology Unit, Fondazione IRCCS Istituto Nazionale dei Tumori, 20133 Milan, Italy; stefano.cavalieri@istitutotumori.mi.it

**Keywords:** normal tissue reaction, particle therapy, autoimmune disease, toxicity

## Abstract

**Simple Summary:**

The decision to offer radiation therapy for cancer in patients with autoimmune diseases is problematic, due to the possibility that such diseases can predispose patients to higher acute and late treatment toxicity by triggering a pro-inflammatory cascade. Specifically, no data are available regarding the impact of this problem on particle therapy. Although the number of patients who access particle therapy is lower than photon treatment, and despite the fact that autoimmune diseases are not a frequent comorbidity in the population, our study reports an increase in terms of acute G3 toxicity in patients with autoimmune diseases compared to a control group without ADs. Since no severe G4–G5 events were reported and in consideration of the benefit of particle therapy for selected cancers, we conclude that article therapy should be not discouraged for patients with autoimmune conditions.

**Abstract:**

It is unclear whether autoimmune diseases (ADs) may predispose patients to higher radiation-induced toxicity, and no data are available regarding particle therapy. Our objective was to determine if cancer patients with ADs have a higher incidence of complications after protons (PT) or carbon ion (CIRT) therapy. METHODS. In our retrospective monocentric study, 38 patients with ADs over 1829 patients were treated with particle therapy between 2011 and 2020. Thirteen patients had collagen vascular disease (CVD), five an inflammatory bowel disease (IBD) and twenty patients an organ-specific AD. Each patient was matched with two control patients without ADs on the basis of type/site of cancer, type of particle treatment, age, sex, hypertension and/or diabetes and previous surgery. RESULTS. No G4–5 complications were reported. In the AD group, the frequency of acute grade 3 (G3) toxicity was higher than in the control group (15.8% vs. 2.6%, *p* = 0.016). Compared to their matched controls, CVD–IBD patients had a higher frequency of G3 acute complications (27.7 vs. 2.6%, *p* = 0.002). There was no difference between AD patients (7.9%) and controls (2.6%) experiencing late G3 toxicity (*p* = 0.33). The 2 years disease-free survival was lower in AD patients than in controls (74% vs. 91%, *p* = 0.01), although the differences in terms of survival were not significant. CONCLUSIONS. G3 acute toxicity was more frequently reported in AD patients after PT or CIRT. Since no severe G4–G5 events were reported and in consideration of the benefit of particle therapy for selected cancers, we conclude that particle therapy should be not discouraged for patients with ADs. Further prospective studies are warranted to gain insight into toxicity in cancer patients with ADs enrolled for particle therapy.

## 1. Introduction

Patients with autoimmune diseases (ADs) represent a challenging clinical scenario when radiation therapy (RT) is recommended for cancer care. ADs are a heterogeneous group of diseases characterized by immune system dysregulation and the development of autoantibodies [1]. Some ADs are systemic, e.g., collagen vascular (CVD) and inflammatory bowel diseases (IBD), and others are organ-specific, e.g., autoimmune thyroiditis, vitiligo, etc. Although the current evidence is not overwhelming, some investigators have reported that immune system defects/modulation possibly affect radiation tolerance, with a higher incidence of acute/late toxicity in cancer patients with ADs receiving RT [2,3,4].

Recently, a systematic review and meta-analysis of 18 studies showed that AD patients have a 10–15% risk of acute and late severe toxicity after photon-based RT [5] compared to the no AD population. These toxicity rates were lower compared to the data reported in previous publications [6,7,8,9], leading to the conclusion that ADs (and especially CVD and IBD) are not absolute contraindications to RT. Nevertheless, a cautious approach for this patient category seems to be reasonable [5]. In addition, it should not be disregarded that, besides modern RT techniques, such as intensity-modulated and stereotactic ablative RT, most of the studies included in the meta-analysis involved now obsolete technologies, for example, two-dimensional RT with delivery of an unwanted dose bath to the tissue surrounding the tumor target.

Particle therapy delivered with protons or carbon ions has physical advantages over conventional photon RT, by depositing the majority of the beam energy at the site of the “Bragg Peak”, with no dose beyond it. Hence, normal tissues distal to the Bragg peak can be protected by avoiding unnecessary radiation doses [10,11].

So far, data about toxicity in patients with ADs treated with particle therapy are not available. In consideration of the immunogenic effect, more pronounced in the high linear energy transfer (LET) component of the particle radiation beam, indeed this issue might be relevant especially for carbon ions [12]. Due to the longer life expectancy and the growing number of patients treated with particle therapy [13,14], the toxicity of proton (PT) or carbon ion therapy (CIRT) in cancer patients with ADs, particularly CVD and IBD, should be better elucidated, which motivates the present study.

## 2. Materials and Methods

### 2.1. Study Design and Inclusion Criteria

This is a retrospective, monocentric, observational matched-pair case–control study, aimed at determining whether patients with ADs and treated for cancer with PT and/or CIRT have a greater risk of grade (G) ≥ 3 acute and late toxicity than subjects without ADs.

We reviewed the institutional patient registry, collecting data on 1829 patients treated with PT and/or CIRT from September 2011 to December 2019 at our institution. Patient inclusion criteria for our study were: diagnosis of ADs (in ADs cohort), age > 18 years, histological or radiological diagnosis of cancer, Karnofsky performance status > 60, minimum follow-up of 6 months after the end of RT and particle therapy with curative intent. Exclusion criteria were: incomplete information about ADs or diagnosis of ADs after receiving PT or CIRT (in ADs cohort), lacking information about treatment-related toxicity, administration of systemic cancer therapy and/or previous RT. 

Cancer patients with ADs were matched on a one-to-two basis with subjects without ADs (control group) pooled out from our institutional registry. The match was performed on the basis of: type of particle therapy (PT, CIRT, PT + CIRT), total radiation dose delivered per RT course (±10 GyRBE) and fractionation, treatment site, age (±10 years), sex and comorbidities (hypertension and diabetes mellitus).

For ADs patients with more than one matching control, the controls with the smallest differences concerning radiation dose and age were chosen, prioritized on the type of particle, treatment fractionation and sex. 

Study patients provided signed informed consent for data processing for research purposes. This study was approved by the local ethics committee (number CNAO-OSS-18-2020). The study was performed in accordance with the ethical standards laid down in the 1975 Declaration of Helsinki and all subsequent revisions.

### 2.2. Treatment Data

Details on target volume delineation, particle therapy planning and PT or CIRT delivery procedures have been previously reported [15,16,17]. In brief, a simulation computed tomography (CT) scan (2 mm slice thickness) was performed for treatment plan calculation and registered with diagnostic contrast-enhanced magnetic resonance imaging (MRI) for rigid anatomy matching. Gross tumor volume (GTV) consisted of macroscopic disease detected at imaging; clinical target volume (CTV) was generated by adding a 0–10 mm margin depending on the location and histology of the primary tumor and further modified based on the possible anatomical tumor-specific spreading pathway (nerves, soft tissues, meninges). Then, a 2–5 mm expansion to the CTV according to the site of the tumor was generated to create the planning target volume (PTV) [15,16,17]. Doses and volumes of particle treatments were defined according to the institutional clinical internal guidelines or protocols specific for disease site, histology and clinical tumor setting.

### 2.3. Follow-Up and Toxicity Evaluation

During treatment, each patient was examined by the dedicated radiation oncologist at the beginning of the treatment and once a week after that, and the toxicity data were collected in the personal patient chart. After the end of treatment, each patient was monitored and followed up according to our institutional policy, and acute and late effects were recorded at each follow-up visit. To evaluate tumor response, MRI or CT were performed every 3–4 months for the first 2 years, and every 6 months afterwards, according to the disease site, histology and treatment clinical setting. Patients with ADs were strictly monitored, together with their general practitioner or immunologist, to evaluate the opportunity for more intensive supportive measures if necessary. Acute (onset within 6 months after treatment) and late (occurring at least 6 months after the end of particle therapy) toxicity were scored according to the Common Terminology Criteria for Adverse Events (CTCAE) v5 [18]. 

### 2.4. Endpoints

The study was designed with the primary objective to compare CTCAE grade ≥3 (G ≥ 3) acute toxicity in ADs patients versus patients without ADs, treated with particle therapy at radical doses. The secondary endpoints of the study were: G ≥ 3 late toxicity profiles among the study groups, incidence of AD reactivation based on the need of starting or modifying immunosuppressive treatment or the need of hospitalization for ADs, difference in acute and late high-grade toxicity among specific ADs (CVC-IBD vs. the remaining ADs), overall survival (OS) and disease-free survival (DFS). 

### 2.5. Statistical Analysis

Median value and range were calculated for continuous variables, and percentages for categorical variables, for ADs patients (cases) and patients without ADs (controls). Accordingly, the Chi-squared test, Fisher Exact test, Mantel–Haenszel test (for trend) and Wilcoxon rank test were used to assess differences between groups of patients in the distribution of categorical, ordinal and continuous variables. Conditional multivariable logistic models were applied to investigate factors associated with acute and late toxicity (age, sex, type of particle, radiation dose, comorbidities, surgery). Survival probabilities over time for DFS and OS were estimated by the Kaplan–Meier method, and the univariate analyses to assess the differences between survival curves of different groups of patients were carried out by the log-rank test. Two-sided *p*-values (*p*) < 0.05 were considered statistically significant. Statistical analyses were performed using the SAS statistical software and R.

## 3. Results

### 3.1. Study Population 

As expected, there was no statistically significant difference among all considered variables in the two groups of patients (type of particle therapy, total dose, GTV, CTV, age, sex, comorbidities, surgery) (Table 1). 

Overall, 38 patients with ADs over 1829 treated patients fit our inclusion criteria. Of these, 12 patients had systemic CVD (5 rheumatoid arthritis, 4 systemic lupus erythematosus, 1 psoriatic arthritis, 1 Sjogren’s syndrome, 1 scleroderma), 5 IBD diseases (3 ulcerative colitis, 1 Crohn’s disease, 2 nonspecific IBD) and 20 patients had a diagnosis of organ-specific ADs (11 autoimmune thyroid diseases, 8 localized skin diseases, 1 genetic autoimmune lymphoproliferative disease). CVD–IBD patients were all in remission phase. The control group included 76 patients. 

Overall, 60 (52.6%) patients were treated for tumors localized at the head and neck (majority originating form salivary glands or sinonasal cavity), 42.1% at the skull base or brain and 6 patients in the pelvis.

Sixty patients received PT at a median dose of 66 GyRBE (range, 50.4–74 GyRBE); the most frequent fractionation scheme was 1.8–2 GyRBE dose per fraction. CIRT was prescribed for 42 patients at a median dose of 67.6 GyRBE in 16 fractions with a median dose per fraction of 4.3 GyRBE (range, 4–4.8 GyRBE). Six patients received a mixed beam approach, including both PT and CIRT, with a median total dose of 75 GyRBE (range 74–75 GyRBE).

### 3.2. Acute Toxicity

For the total group of 114 patients, toxicity was scored G0–G1 in 59 cases (51.8%), G2 in 50 (41.2%) and G3 in 8 (7%) cases. No G4–5 complications were reported. 

Radiation dermatitis (*n* = 3) and oral mucositis (*n* = 4) were the most common G3 toxicities. One patient developed G3 middle ear inflammation (mastoiditis). No treatment modifications or interruptions were reported because of acute toxicity events. All patients with G3 toxicity had a complete resolution of symptoms within 6 months after the end of treatment. 

In 50 patients with G2 acute toxicity, 74 G2 events were reported. Skin and mucosal toxicity were the most common G2 acute toxicities: radiation dermatitis (*n* = 25 patients), oral mucositis (*n* = 35) and alopecia (*n* = 2). Other G2 events were: dysphagia (*n* = 5), middle ear information (*n* = 2), neuralgia (*n* = 2), conjunctivitis (*n* = 2) and headache (*n* = 1). None of these toxicities occurred more frequently in the ADs group than in the control group.

The frequency of G3 toxicity was higher in patients with ADs than in those without (Table 2). A G3 acute toxicity was observed in six cases and two controls (15.8% vs. 2.6%, respectively; *p* = 0.016).

Compared to their control group counterparts, CVD–IBD patients experienced a higher G3 acute toxicity rate (*p* = 0.002) (Table 3). The rate of acute G3 toxicity in the CVD–IBD subgroup was 27.7%. Organ-specific ADs patients did not report more events of severe acute toxicity (*p* = 0.164).

GTV correlated well with the occurrence of G3 toxicity as shown in Table 4, while no correlation was found between CTV and acute toxicity (*p* = 0.28). Age, type of particle, radiation dose, the presence/absence of comorbidities and previous surgery were not associated with acute G3 toxicity in the two groups, as shown in Table 5. 

Considering the occurrence of acute toxicity of G0 vs. G ≥ 1, no significant difference between AD patients and controls was observed (*p* = 0.31).

### 3.3. Late Toxicity

Overall, 80 (70.2%) patients developed late G0–G1 toxicity, 29 (25.4%) late G2 and 5 late G3 (4.4%) toxicity. No G4–5 late events were recorded. 

The G3 late events—hearing impairment (*n* = 3), oral mucositis (*n* = 1) and central nervous system necrosis (*n* = 1)—occurred after a median time of 13 months (range, 6–26 months) after the end of particle therapy. Two patients (one in the control cohort and one in the ADs) with G3 hearing impairment had G3 acute toxicity (mucositis and dermatitis) during treatment. 

The patients with late G3 toxicity (Table 2) were three cases and two controls (7.9% vs. 2.6%, respectively; *p* = 0.33). The occurrence of G3 late toxicity was at 15 and 26 months after the end of therapy in the two controls, and from 6 and 13 months for the AD patients.

Thirty-eight cases of G2 late toxicity were reported in 29 patients: localized edema and soft tissue fibrosis (*n* = 8), xerostomia (*n* = 7), neuralgia (*n* = 6), hearing impairment (*n* = 5), cranial nerve disorders (*n* = 4), trismus (*n* = 3), tinnitus (*n* = 2), alopecia (*n* = 1), central nervous system necrosis (*n* = 1) and hypopituitarism (*n* = 1). None of these toxicities occurred more frequently in the ADs group than in control group. 

When the analysis was focused on the specific AD subgroup (CVD–IBD or organ-specific ADs), none of the AD subgroups showed a statistically significant difference from their respective controls. Furthermore, we did not find any correlation between the occurrence of late toxicity in the two groups and age, type of particle treatment, radiation dose, the presence/absence of comorbidities and previous surgery (Table 5). No correlation was found between GTV (*p* = 0.75) and CTV (*p* = 0.98) and late toxicity.

No significant difference between AD and controls (*p* = 0.66) was reported in the occurrence of late toxicity of G0 vs. G ≥ 1.

### 3.4. AD Reactivation

Two patients (5.2%) experienced a reactivation of their ADs (IBD in both cases) during or after treatment.

A 33-year-old woman with ulcerative colitis treated with PT for a salivary gland tumor required hospitalization during treatment for aggravation of ulcerative colitis. A colonoscopy was performed with a diagnosis of reactivation. Anti-inflammatory therapy led to transient clinical relief that made possible the conclusion of PT, but PT was interrupted for 2 days. Twelve days after the end of the treatment, she underwent a total colectomy for colonic ischemia. After 12 months follow-up, she was in good clinical conditions and without local or distant oncologic relapse. She did not report acute or late toxicity G ≥ 3. 

A 37-year-old man treated for a skull-base chordoma had a previous diagnosis of IBD (Crohn’s disease). During his clinical history, he had received anti-tumor necrosis factor (TNF) therapy (adalimumab), which was stopped 4 months before PT. After 6 months, due to the progression of IBD-related symptoms, he started a new therapy with monoclonal antibody (ustekinumab). After 15 months of follow-up, chordoma was stable and Crohn’s disease was under control. 

### 3.5. Survival 

**Figure 1:**
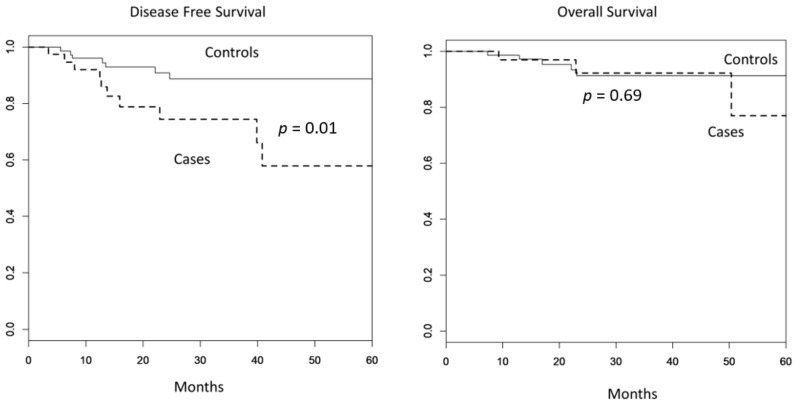
Disease-free survival (DFS) and overall survival (OS) in cases (ADs) and controls (no ADs). DFS at 2 years in cases: 74%, DFS at 2 years in controls: 91% (*p* = 0.01). OS at 2 years in cases: 92%, OS at 2 years in controls: 91% (*p* = 0.69).

Median follow-up time was 30 months (range, 7–79.4 months) in the total population under investigation. Patients with ADs had a shorter 2-year DFS (Figure 1) than the controls (74% vs. 91%, *p* = 0.01). The 2-year OS was not significantly different for patients with and without ADs (92% vs. 91%, *p* = 0.69).

## 4. Discussion

To our knowledge, this is the first matched case–control study aiming to describe the acute and late toxicity profile of particle therapy in cancer patients with ADs. So far, the data available about the impact of ADs on toxicity are mainly derived from the experience with photon RT. The most comprehensive systematic review and meta-analysis on the issue of ADs and RT included 621 patients with CVD and 204 with IBD, from 18 series treated with conventional photon RT. In this meta-analysis, an incidence was estimated of acute and late G ≥ 3 toxicity in CVD patients of 11.7% and 6.1%, respectively, and in IBD patients of 14.0% and 10.2%, respectively [5]. Overall, G4 acute and late toxicity events were 1.5% and 4.5%, respectively, and G5 toxicity was negligible (<1%) [5]. 

Focusing on matched cohort studies only (Table 6), one of which was included in the previous meta-analysis [19], the percentage of G ≥ 3 acute and late toxicities in the AD cohorts varied from 7% to 14% and from 7% to 17%, respectively [19,20,21,22]. Overall, no difference was found in terms of severe acute toxicity among patients with ADs—all of them with CVD—and the control groups. On the other hand, considering the late side effects, Chen et al. reported a significant difference in patients with CVD compared to controls (17% vs. 3%, *p* = 0.0095) [20], and a trend towards an increased rate of G ≥3 late toxicity (9.3% vs. 3.7%, *p* =0.079) was found in the paper by Lin et al. [22]. Additionally, a recent single-institution retrospective study on 194 patients with CVD treated for cancer reported that a severe acute and late toxicity profile was not correlated with the different RT schedules and dose fractionation regimens (conventional fractionation, moderate hypofractionation and ultra-hypofractionation) [23].

Considering these data derived from the photon-based RT literature, it could be concluded that patients with ADs, especially CVD and IBD, experienced acceptable rates of toxicity, although with variable ranges, related to the heterogeneity of irradiation site and dose, clinical setting and use of concomitant chemotherapy. In our case–control pair-matched study after particle therapy, the frequency of G3 acute toxicity in patients with ADs was significantly higher than in subjects without ADs. In particular, in patients with CVD–IDB, acute G3 toxicity was reported in 27.7% of cases. Furthermore, G3 late toxicity was observed in 7.9% of ADs cases, without any significant difference with the control patients. Any G4 and G5 toxicity was not reported, neither in the case nor in the control group.

In the following, we highlight the peculiarity of our study compared with the matched cohort studies with photon RT cited before, and discuss the results of our analysis. First of all, we considered a broad spectrum of autoimmune disorders, some of which are not listed in the meta-analysis and matched cohort series mentioned before (such as the organ-specific ADs most frequently reported at our institution) in order to not disregard their possible impact on the risk of severe toxicity. Concerning this issue, we point out that the inclusion of seven patients with localized autoimmune skin diseases (psoriasis and vitiligo) could have had an impact on the high rate of severe dermatitis reported. Indeed, psoriasis is reported to involve an upregulation of various immune cells (plasmacytoid dendritic and T helper cells), as well as the release of key cytokines such as TNF-a, interleukin-23 (IL-23), IL-17 and IL-22 [24]. Furthermore, in our analysis we also included patients with thyroid autoimmune diseases, in view of the recent publications showing that the chronic upregulation of inflammatory mediators and cytokines plays a key role in the onset of these disorders [25,26]. Although our study considered organ-specific ADs that were not always reported in the previous literature on photon RT, we confirmed that the type of AD most frequently associated with high-grade radiation-induced toxicity is systemic disorders (CVD–IBD).

Differently from previous publications, our study reported on patients treated with PT or CIRT. It is well known that particles have dosimetric advantages over photons due to their peculiar depth–dose profile, delivering lower doses to normal tissues outside the tumor target. In fact, the rate of acute and late toxicity in the control group in our series of patients was lower compared to the published series (Table 5). In light of their peculiar physical properties, it could therefore be speculated that particles can provide a lower incidence of toxic effects in AD patients as well, as compared to photon RT. Considering protons, preclinical radiobiology studies reported a tendency towards a reduced inflammatory response in normal tissues when compared to photons. However, further investigations are needed in order to support these preliminary findings [27]. For carbon ions, preclinical data on the issue are conflicting. While in some research studies carbon ions seem to be able to trigger inflammatory signaling and accelerate the differentiation of keratinocytes to a similar extent as photons at the same doses [28], more recent data reported that high doses of CIRT may be proinflammatory, inducing especially the production of granzyme B, IL-2 and TNF-a [29]. However, how these tissue-specific effects can be related to the burden of toxicity is unclear. Surprisingly, the differential choice of PT or CIRT in our series did not affect acute and late toxicity, and the potential higher immunogenicity of CIRT was not related to more complications in patients with an immune system dysregulation such as ADs. In this regard, the straightforward implication is that even the hypofractionated CIRT schedules, compared to the conventional fractionation schemes of PT in our series, did not have an impact on the late toxicity profile, confirming the result on the independence of fractionation reported for conventional RT [23].

Among the matched control studies previously mentioned, two of them reported on toxicity by irradiation site [19,22]. On the contrary, here in our study, we reported toxicity data on the cohort of patients together with different irradiation sites. In our cohort, all patients received particle therapy with curative intent, with high radiation dose, and the majority of patients had head and neck cancers with a large amount of skin and mucosa included in RT volumes; thus, radiation dermatitis and oral mucositis were the most common G3 acute toxic events. Similarly, although thorax and pelvis were the most irradiated sites, in Ross et al. the most common acute reaction was confluent mucositis occurring in patients treated for head and neck cancers [19]. Even in Lin et al., RT in CVD patients produced higher crude rates of acute G3 skin toxicity with severe desquamation in breast or pelvis irradiation [22].

Concerning late toxicity, in contrast with the previous series, we did not find any difference in G3 late toxicity between control and ADs patients, although we reported an earlier onset of severe toxicity after the end of RT in the ADs group. Moreover, the analysis performed on patients treated with exclusive PT or CIRT treatment (without surgery) or with particle therapy after surgery did not show a significant difference in terms of late toxicity, although studies in which the combined treatment (surgery + RT) is burdened by major toxicities have been reported in the literature [30,31]. Finally, differently from other studies [32], neither G4 (e.g., skin/soft-tissue necrosis or ulceration) nor treatment-related deaths were observed in our cohort of patients, thus underlying the intrinsic physical characteristics of the PT and CIRT in sparing healthy tissues surrounding the target. 

In our opinion, these results should be interpreted with caution, especially in consideration of the median follow-up time of our analysis, which was shorter compared to the previous series investigating conventional RT. In addition, currently, no exhaustive data are available on the relationship between radiobiological properties at the cellular and tissue levels after particle RT (e.g., DNA damage and inflammatory response, changes in lymphocyte counts, downregulation of angiogenesis and cell migration pathways), and late side effects. In our study, ADs patients had a lower 2-year DFS than controls. In the literature, previous studies on photon RT in patients with ADs did not always focus on survival outcome and disease control. Chen et al. reported that patients with CVD had no statistical differences in OS and DFS compared to their matched control subjects [20]. Concerning CIRT, it has been shown that it induces antitumor immunity in immunocompetent animals. Therefore, if we consider CIRT as an immune-stimulatory agent, we should have expected a higher effect also in the AD treated cases where the presence of an unbalanced and dysregulated immune system could elicit an immune response against cancer. Although in our series, only for one patient did we have a longer overall treatment time for AD reactivation, in general, it could be hypothesized that the discontinuation of RT treatment in some cases or the assumption of immunosuppressive agents before or during the AD patient clinical history could affect treatment response and LC. Nevertheless, the literature lacks data, and more information is needed to explain and confirm the difference of treatment response in AD patients.

In this context, and especially for CIRT, the immunomodulation effect on distant metastatic sites besides the local effect (abscopal effect) has been reported [33]. Since no data are available on the impact of ADs, we believe that in the future the issue of dysregulation of the immune system in ADs and the abscopal effect should be further investigated.

As for previous publications on the same issue, due to the rarity of ADs in the general population, it can be argued that our study has some methodological limitations, such as the retrospective nature and the limited sample size. The relative scarcity of cancer patients with ADs, the heterogeneous cancer histology and radiation treatment site (head and neck cancers are more represented than pelvic cancers) necessarily affect the scientific impact of retrospective studies. In addition, most of the previous series on this topic, including three of the matched cohort studies reported in Table 6, considered patients with different characteristics in terms of treatment site, radiation technique and aim of the treatment.

Furthermore, due to the limited number of AD patients receiving particle therapy, a large-scale patient enrolment is difficult for a single-center study, possibly suggesting for the future an initiative of cooperation between multiple particle therapy institutions to collect larger cohorts of ADs patients to be investigated in prospective trials. Furthermore, prospective studies would allow translational research, for example, on circulating lymphocytes differently activated in AD patients than in non-AD patients.

## 5. Conclusions

Our study showed higher acute toxicity in cancer patients with ADs treated with particle therapy at our institute compared to the control group, despite the low incidence of severe late toxicity compared to the published series and no life-threatening events. Particle therapy should not be withheld from AD patients with cancer, but caution is required. In our series, patients treated with CIRT had no higher toxicity compared to patients receiving PT treatment. In consideration of the different DFS in the AD and control groups in our series, further studies are needed to dissect the role of particle therapy and immune regulation in the case of cancer patients affected by ADs.

## Figures and Tables

**Table 1:** Patient characteristics.

	Total *n* = 114 (100%)	Cases *n* = 38 (100%)	Controls *n* = 76 (100%)	*p*-Value
Age, years				0.99
Median	56	56	57	
Range	20–81	20–77	23–81	
Sex, *n* (%)				1.00
Female	93	31	62	
	(81.6)	(81.6)	(81.6)	
Male	21	7	14	
	(18.4)	(18.4)	(18.4)	
Particle, *n* (%)				1.00
P	66	22	44	
	(57.9)	(57.9)	(57.9)	
C	42	14	28	
	(36.8)	(36.8)	(36.8)	
P + C	6	2)	4	
	(5.3)	(5.3	(5.3)	
Radiation dose GyRBE				
PT				
Median	66	63	66	
range	50.4–74	50.4–74	50.4–74	0.18
CIRT				
Median	67.6	66.7	67.6	
range	64–76.8	64–73.6	65.6–76.8	0.20
PT + CIRT				
Median	75	75	75	
Range	74–75	75–75	74–75	0.45
Site of tumor, *n* (%)				1.00
Skull base	48	16	32	
	(42.1)	(42.1)	(42.1)	
Head and neck	60	20	40	
	(52.6)	(52.6)	(52.6)	
Pelvis	6	2	4	
	(5.3)	(5.3)	(5.3)	
GTV, cm^3^				0.6
Median	10.1	8.7	11.1	
Range	(0–507)	(0–218.6)	(0–507)	
CTV, cm^3^				0.74
Median	53.1	51.4	54.9	
Range	(0.24–2802)	(3.7–650.8)	(0.24–2802)	
Comorbidity, *n* (%)				0.10
No	83	24	59	
	(72.8)	(63.2)	(77.6)	
Yes	31	14	17	
	(27.2)	(36.8)	(22.4)	
Surgery, *n* (%)				1.00
No	30	10	20	
	(26.3)	(26.3)	(26.3)	
Yes	84	28	56	
	(73.7)	(73.7)	(73.7)	

PT: proton therapy; CIRT: carbon ion therapy; *p*-value: Chi-squared test or Wilcoxon rank test.

**Table 2:** High-grade toxicity in case (ADs) and control (no ADs) groups.

		Total *n* = 114 (100%)	Cases *n* = 38 (100%)	Controls *n* = 76 (100%)	*p*-Value
Acute G3 toxicity	No	106 (93.0%)	32 (84.2%)	74 (97.4%)	0.016
	Yes	8 (7.0%)	6 (15.8%)	2 (2.6%)	
Late G3 toxicity	No	109 (95.6%)	35 (92.1%)	74 (97.4%)	0.33
	Yes	5 (4.4%)	3 (7.9%)	2 (2.6%)	

ADs: autoimmune diseases; *p*-value: Fisher Exact test.

**Table 3:** High-grade toxicity in CDV-IBD and ADs other than CDV-IBD patients.

			*p*-Value
	CDV-IBD *n* = 18	Controls *n* = 36	
Acute toxicity G3 Late toxicity G3	5 (27.78%)	2 (2.63%)	0.002
	2 (11.11%)	2 (2.63%)	0.164
	ADs other than CDV-IBD *n* = 20	Controls *n* = 40	
Acute toxicity G3 Late toxicity G3	1 (5%)	2 (2.63%)	0.508
	1 (5%)	2 (2.63%)	0.508

CVD: collagen vascular disease; IBD: inflammatory bowel disease; ADs: autoimmune diseases; *p*-value: Fisher Exact test.

**Table 4:** Associations of grade 3 acute and late toxicity with GTV.

	Total *n* = 114	Median GTV (cm^3^)	*p*-Value
Acute toxicity G0 G1 G2 G3	21 (18%) 35 (31%) 50 (44%) 8 (7%)	8.4 12.4 8.4 14.9	<0.0001

*p*-value: from Wilcoxon rank test.

**Table 5:** Associations of grade 3 acute and late toxicity with clinical variables.

Particle	CIRT (*n* = 42)	PT (*n* = 66)	PT + CIRT (*n* = 6)	*p* Adjusted for Case–Control
Acute G3	4 (9.5%)	3 (4.6%)	1 (16.7%)	0.407
Late tox G3	3 (7.1%)	1 (1.5%)	1 (16.7%)	0.207
**Age**	**<56 years (*n* = 58)**	**≥56 years (*n* = 56)**		
Acute G3	4 (6.9%)	4 (7.1%)		0.906
Late tox G3	0 (0%)	5 (8.9%)		0.994
**Dose CIRT**	**<67.6 GyRBE (*n* = 21)**	**≥67.6 GyRBE (*n* = 21)**		
Acute G3	1 (4.8%)	3 (14.3%)		0.326
Late tox G3	1 (4.8%)	2 (9.5%)		0.566
**Dose PT**	**<66 GyRBE (*n* =32)**	**≥66 GyRBE (*n* = 34)**		
Acute G3	4 (6.9%)	4 (7.1%)		0.551
Late tox G3	2 (6.3%)	1 (2.9%)		0.997
**Comorbidity**	**No (*n* = 83)**	**Yes (*n* = 31)**		
Acute G3	4 (4.8%)	4 (12.9%)		0.289
Late tox G3	3 (4%)	2 (6.5%)		0.657
**Surgery**	**No (*n* = 30)**	**Yes (*n* = 84)**		
Acute G3	1 (3.3%)	7 (8.3%)		0.365
Late tox G3	0 (0%)	5 (5.9%)		0.995

PT: proton therapy, CIRT: carbon ions therapy; *p*-value: from conditional logistic model.

**Table 6:** Matched control studies.

Study	ADs	Number of Patients	RT Treatment	Site of Irradiation	Aim of RT	Follow-Up	Acute ≥ G3 Toxicity	Late ≥ G3 Toxicity	Fatal Events
Ross et al. [19]	CVD	Control: 61 CVD: 61	EBRT, brachytherapy, EBRT + brachytherapy	Thorax: 26% Pelvis: 23% Head and neck: 13% Breast: 10% Others	Both curative and palliative	-	Control: 7% CVD: 11% (*p* = 0.3)	Control: 7% CVD: 10% (*p* = 0.6)	3 G5 toxicity in CVD group
Chen et al. [20]	CVD	Control: 36 CVD: 72	EBRT	Breast: 100%	Adjuvant	Mean 12 years	Control: 8% CVD: 14% (*p* = 0.4)	Control: 3% CVD: 17% (*p* = 0.009)	No G5 toxicity
Phan et al. [21]	CVD	Control: 38 CVD: 38	EBRT, brachytherapy	Breast: 34% Gynecologic: 28% Head and neck: 7% Lung: 7% Prostate: 7% Others	Both curative and palliative	Median 36 months (curative) 3 months (palliative)	Control: 7% CVD: 7%	Control: 7% CVD: 7%	No G5 toxicity
Lin et al. [22]	CVD	Control: 222 CVD: 86	EBRT	Thorax: 18% Skin: 16% Head and neck: 14% Pelvis: 13% Breast: 9% Others	-	Median 1.3 years	Control: 10% CVD: 10 %	Control: 3.7% CVD: 9.3 % (*p* = 0.079)	1 G5 toxicity in CVD group
Our study	CVD–IBD + organ specific ADs	Control: 76 ADs: 38	PT CIRT	Head and neck: 52.6% Brain and skull base: 42.1% Pelvis: 5.3%	Curative	Median 30 months	Control: 2.6% ADs: 15.8% (*p* = 0.016)	Control: 2.6% ADs: 7.9% (*p* = 0.33)	No G5 toxicity

ADs: autoimmune diseases; CVD: collagen vascular disease; IBD: inflammatory bowel disease; RT: radiotherapy; EBRT: external beam photon-based radiotherapy; PT: proton therapy; CIRT: carbon ion therapy.

## Data Availability

Data sharing not applicable.
